# Sleeve resection with end-to-end anastomosis in the reconstruction of tracheal defects exceeding six rings: a clinical feasibility study and safety assessment

**DOI:** 10.3389/fsurg.2023.1229522

**Published:** 2024-04-12

**Authors:** Xin Xia, Xiaoli Zhu, Yingying Zhu, Wenwen Diao, Xingming Chen

**Affiliations:** Department of Otolaryngology-Head and Neck Surgery, Peking Union Medical College Hospital, Peking Union Medical College and Chinese Academy of Medical Sciences, Beijing, China

**Keywords:** tracheal defect, long-segment, tracheal reconstruction, sleeve resection, end-to-end anastomosis

## Abstract

**Objectives:**

Reconstruction is always required for tracheal defects and sleeve resection with end-to-end anastomosis is the most common used. The aim of the study was to present surgical techniques and evaluate the outcomes of sleeve resection with end-to-end anastomosis in the reconstruction of tracheal defects exceeding six rings.

**Methods:**

The study included patients with primary or secondary malignancies and tracheal stenosis from 2014 to 2019, who were treated with sleeve resection exceeding six tracheal rings, and reconstructed with end-to-end anastomosis. Airway status and patient outcomes were the principal follow-up measures.

**Results:**

A total of 16 patients were enrolled in the study including three primary tracheal malignancies, 12 invasive thyroid carcinomas and one with tracheal stenosis. The extent of tracheal resection ranged from seven to nine rings, and the primary end-to-end anastomosis was performed in all 16 patients. Performance of tracheostomy or cricothyroidotomy was done in 6 patients with decannulation at a median of 42 days (range, 28–56). No anastomotic dehiscence, infection or bleeding occurred postoperatively, and all 16 patients maintained an unobstructed airway through the end of follow-up.

**Conclusions:**

Sleeve resection reconstructed with end-to-end anastomosis can serve as an appropriate therapeutic strategy for the tracheal defects even exceeding six rings. Adequate laryngeal release is the key to surgical success.

## 1 Introduction

Resection of primary or secondary tracheal malignancies with intraluminal invasion and advanced stenosis results in significant tracheal defects. Reconstruction is always required, which typically requires sleeve resection with end-to-end anastomosis or window resection with flap repair. To avoid lumen instability and stenosis, window resection with flap reconstruction is applicable only for defects of less than one-half the circumference (1). For more extensive circumferential defects, sleeve resection with primary anastomosis is necessary and is associated with a high success rate and minimal morbidity (2, 3).

However, anastomotic dehiscence is the most feared complication of sleeve resection with primary anastomosis, primarily relating to length of the resected segment and tension on the anastomosis. Prior studies have reported that a maximum of six tracheal rings can be resected and safely anastomosed with acceptable tension (4). Although achieving a primary secure anastomosis can pose a significant challenge following a long-segment (over six tracheal rings) resection, it should be undertaken whenever possible to optimize the patient's quality of life.

This retrospective study describes the surgical techniques of sleeve resection with primary end-to-end anastomosis for the reconstruction of defects exceeding six tracheal rings, and evaluates the surgical and survival outcomes in a cohort of 16 patients.

## 2 Materials and methods

### 2.1 Study participants

This was a retrospective analysis comprised of patients with primary tracheal tumors, invasive thyroid malignancies with invasion into the trachea, and tracheal stenosis, all of whom underwent sleeve resection exceeding six tracheal rings, reconstructed with primary end-to-end anastomosis at Peking Union Medical College Hospital between January 2014 and December 2019.

### 2.2 Clinical parameters

Participant data including sex, age, preoperative examination, treatment, and prognosis were retrieved from medical records. Fiberoptic laryngoscopy and computed tomography were used to evaluate the primary disease, affected airway segments and mobility of the vocal cords. The operative parameters included defect site and size, of which the size was counted by the number of tracheal rings rather than the absolute length. The time to decannulation was recorded if a tracheostomy or cricothyroidotomy was performed. The postoperative parameters included surgical complications (e.g., anastomotic dehiscence, emerging recurrent laryngeal nerve paralysis, local infections, and bleeding), airway condition and disease status at the conclusion of the observation time.

### 2.3 Surgical techniques

For patients with primary or invasive malignancy, the tumor was resected *en bloc* including full-thickness tracheal sleeve resection. If necessary, additional tracheal segments were resected based on intraoperative pathologic frozen section analyses to achieve cancer-free margins. If the stenosis or invasion involved only the trachea inferior to the cricoid cartilage, sleeve tracheal resection including the affected segments was performed. For lesions that were adjacent to the mucosal surface of the anterior cricoid cartilage, the anterolateral cricoid cartilage was resected along with the affected trachea, of which the posterior cricoid plate was preserved.

To achieve an anastomosis with minimal tension, release of the trachea and larynx was conducted. The tracheal release was carried out using blunt dissection. The laryngeal release was performed by transection of the suprahyoid and infrahyoid muscles and removal of the entire hyoid bone. If the mobilization was still not sufficient to achieve a secure anastomosis with acceptable tension, the muscles and ligaments attached to the superior cornu of the thyroid cartilage were further cut off to achieve maximum laryngeal release.

After completion of the sleeve resection and adequate release of trachea and larynx, a primary end-to-end anastomosis was performed with “2-0” continuous polypropylene sutures. The anastomosis was started in the membranous part of the trachea, and continuous sutures, running from the membranous portion to the left and the right cartilaginous portions simultaneously, were placed and fixed by a “2-0” polypropylene with two suture needles. The only one knot of the suture was made outside the anterior tracheal lumen. A tracheostomy was performed as necessary according to anastomotic tension and recurrent laryngeal nerve status for preservation of the airway stability. In extreme cases, a cricothyroidotomy was performed when, following the anastomosis, the remaining trachea was totally below the thoracic inlet. Fiberoptic laryngoscopy was performed to assess the anastomosis and lumen stability immediately upon completion of the operation.

### 2.4 Patient follow-up

Follow-up was conducted regularly using fiberoptic laryngoscopy and imaging. Airway status, locally recurrent malignancy or distant metastasis, and death due to disease or other reasons were recorded as study endpoints. Follow-up continued until at least one of the study endpoints had been reached.

## 3 Results

The general characteristics and clinical parameters of the study participants are summarized in Table 1. A total of 16 patients were enrolled including three primary tracheal malignancies, 12 advanced invasive thyroid carcinomas and one tracheal stenosis case. Tracheal status and vocal cord movement were evaluated preoperatively and results showed nine patients with recurrent laryngeal nerve paralysis including one with bilateral paralysis.

**Table 1:** Characteristics of study participants.

Pat. no.	Sex (F/M)	Age (year)	Disease/pathology	Preoperative RLN status	Range of resection (defects, rings)	Tracheostomy/cricothyroidotomy (decannulation, days)	Survival outcome (months)
1	F	25	TMEC	Normal	1st–7th rings (7)	No	Alive, NED (30)
2	M	68	TACC	Normal	1st–7th rings (7)	Yes (35)	Alive, DM (61)
3	M	61	TACC	Normal	2nd–8th rings (7)	No	Alive, NED (25)
4	M	29	CPTC	U. RLNP	Cricoid, 1st–7th rings (7)^a^	No	Alive, NED (38)
5	M	54	CPTC	U. RLNP	2nd–8th rings (7)	No	Alive, NED (48)
6	F	45	CPTC	Normal	1st–7th rings (7)	No	Alive, NED (29)
7^c^	F	63	CPTC	U. RLNP	Cricoid, 1st–9th rings (9)^a^	No	Alive, NED (39)
8	F	56	CPTC	U. RLNP	1st–8th rings (8)	Yes (28)	Alive, NED (31)
9	M	47	CPTC	U. RLNP	1st–8th rings (8)	No	Alive, NED (43)
10	M	56	CPTC	U. RLNP	2nd–8th rings (7)	No	Alive, NED (41)
11	M	72	CPTC	U. RLNP	3rd–10th rings (8)	Yes (42)	Alive, NED (81)
12	F	53	CPTC	Normal	2nd–8th rings (7)	No	Alive, NED (39)
13	M	64	FVPTC	Normal	1st–7th rings (7)	No	Alive, NED (50)
14	M	39	TSCC	U. RLNP	Cricoid, 1st–9th rings (9)^a^	Yes (49)	Dead, DM (32)
15	M	55	MTC	U. RLNP	5th–13th rings (9)	Yes (42)	Alive, NED (35)
16	F	40	Stenosis (prior operation^b^)	B. RLNP	Cricoid, 1st–8th rings (8)^a^	Yes (56)	Alive, NED (47)

Pat. No., patient number; F, female; M, male; TMEC, tracheal mucoepidermoid carcinoma; TACC, tracheal adenoid cystic carcinoma; CPTC, classical papillary thyroid carcinoma; FVPTC, follicular variant of papillary thyroid carcinoma; TSCC, thyroid squamous cell carcinoma; MTC, medullary thyroid carcinoma; RLN, recurrent laryngeal nerve; U. (B.) RLNP, unilateral (bilateral) recurrent laryngeal nerve paralysis; NED, no evidence of disease; DM, distant metastasis.

^a^ Cricoid, part of the cricoid was resected.

^b^ Prior operation, restenosis after prior window resection and reconstruction with flap.

^c^ 7, the typical case.

Tracheal and cricotracheal sleeve resections were performed in 12 and 4 patients respectively, with vertical defects ranging from seven to nine tracheal rings. Tracheostomy or cricothyroidotomy was performed in 6 patients, and the tubes were decannulated after a median interval of 42 days (range, 28–56). Nasogastric tube intubation was undertaken in all patients to prevent aspiration, and all were removed within two weeks. To achieve breathing independent of tracheostomy, the one patient with preoperative bilateral recurrent laryngeal nerve paralysis underwent additional unilateral vocal cord resection, allowing decannulation 56 days later.

No anastomotic dehiscence, iatrogenic recurrent laryngeal nerve paralysis, local infection or bleeding occurred in any patient postoperatively, with a median follow-up of 39 months (range, 25–81). Airway patency with adequate laryngeal function was observed in all 16 patients at the end of follow-up. Fourteen out of 15 patients, operated for primary or secondary tracheal tumors, received postoperative treatment except the patient with tracheal mucoepidermoid carcinoma. Ten patients with classical papillary thyroid carcinoma or follicular variant of papillary thyroid carcinoma received I^131^ therapy; one medullary thyroid carcinoma patient and two tracheal adenoid cystic carcinoma patients received radiotherapy; the thyroid squamous cell carcinoma patient received chemoradiotherapy. Thirteen out of the 15 patients with primary or invasive malignancy remained disease-free. The patient with tracheal adenoid cystic carcinoma developed pulmonary metastasis 21 months after surgery but was alive 61 months follow-up. The thyroid squamous cell carcinoma patient died of pulmonary and bone metastasis 32 months postoperatively.

### 3.1 Typical case presentation

**Figure 1:**
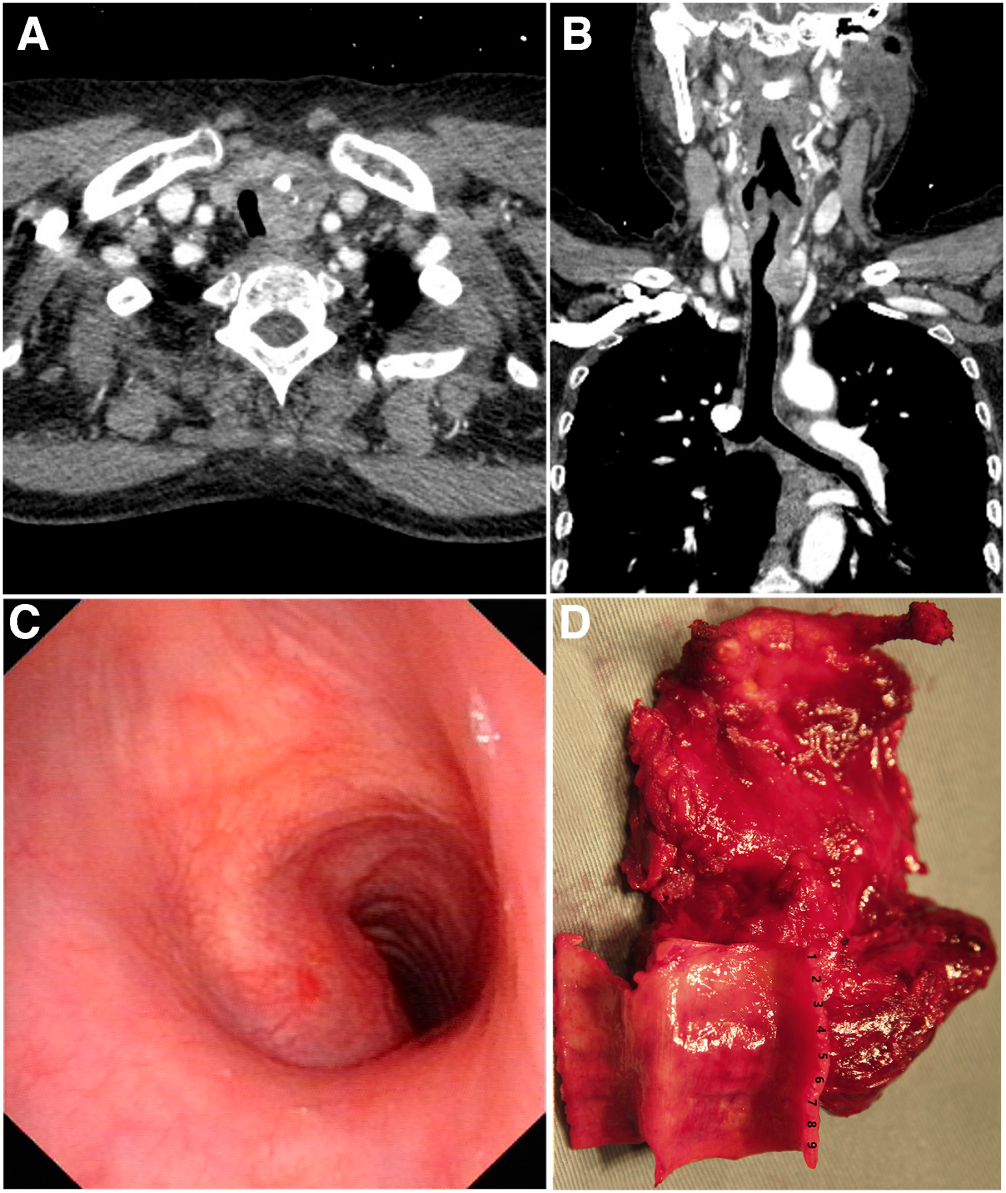
(**A**) and (**B**) computed tomography showed a thyroid neoplasm with invasion of the trachea. (**C**) Fiberoptic laryngoscopy evaluation showed that the tumor invaded the left tracheal wall with obstruction of approximately one-half of the lumen. (**D**) The *en-bloc* resection specimen. The 1st–9th tracheal rings, anterolateral cricoid cartilage, cervical strap muscles and the hyoid bone were resected along with the thyroid carcinoma.

A 63-year-old female was admitted with a 3-month history of hoarseness and dyspnea. Ultrasound detected a hypoechoic irregular mass with unclear borders in the lower left thyroid lobe. Computed tomography confirmed a 2.7 × 2.3 × 3.6 cm mass with inhomogeneous enhancement in the left thyroid lobe with invasion of the 2nd–8th tracheal rings (Figures 1A,B). Preoperative fiberoptic laryngoscopy showed fixation of the left vocal cord and an invasive tumor of the left tracheal wall, obstructing approximately one-half of the lumen (Figure 1C). Fine needle biopsy of the mass showed the histology of classical papillary thyroid carcinoma.

**Figure 2:**
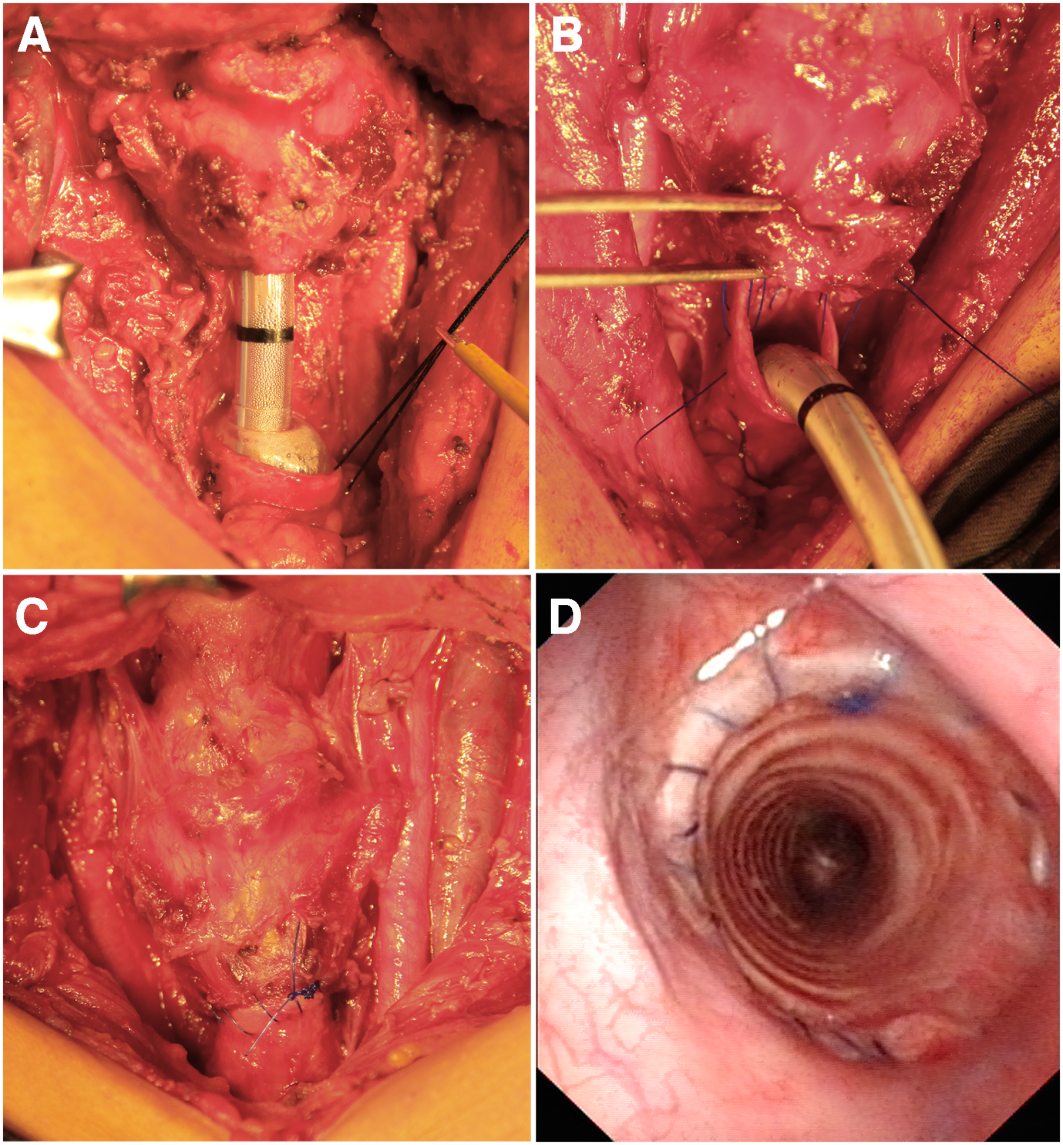
(**A**) Adequate release of the trachea and larynx. (**B**) The end-to-end anastomosis with continuous “2-0” polypropylene sutures. (**C**) Secure larynx-to-trachea anastomosis with minimal tension. (**D**) Fiberoptic laryngoscopy showed stability of the anastomosis and lumen 6 months postoperatively.

At the time of surgery, the cancer was confirmed to invade the left recurrent laryngeal nerve and the 2nd–8th tracheal rings. The cancer was resected *en bloc* as a radical thyroidectomy, including resection of the invaded left recurrent laryngeal nerve, full-thickness sleeve resection of the 1st–9th tracheal rings and anterior of the cricoid cartilage (Figure 1D). Adequate release of trachea and larynx was achieved by tracheal blunt dissection, transection of the suprahyoid and infrahyoid muscles, removal of the hyoid bone and division of the attached muscles and ligaments from the superior cornu of thyroid cartilage (Figure 2A). A primary larynx-to-trachea anastomosis was performed with continuous “2-0” polypropylene sutures under minimal tension (Figures 2B,C). No anastomotic leakage, lumen stenosis or other severe complication occurred postoperatively. Fiberoptic laryngoscopy evaluation six months following the surgery showed the anastomosis and lumen was stable without granulation or stenosis (Figure 2D). No local or systemic tumor recurrence was found at follow-up 39 months postoperatively.

## 4 Discussion

Tracheal reconstruction for large defects is a difficult surgical challenge. The surgical alternatives usually include end-to-end anastomosis following sleeve resection, or reconstruction using a local muscle or perichondral flap after window resection (1). Sleeve resection with end-to-end anastomosis is more appropriate for lesions of the membranous trachea and cases with extensive circumferential defects, whereas flap reconstruction should be considered for defects of less than one-half the circumference to avoid an unstable lumen (5, 6). Additionally, sleeve resection with end-to-end anastomosis provides a full thickness specimen for thorough evaluation of the malignant depth of invasion and margin status, and uncommonly a need for tracheostomy (7).

A secure anastomosis with minimal tension is crucial to the success of an end-to-end reconstruction, because anastomotic dehiscence is the most dreaded, life-threatening complication, typically attributed to excessive tension (8, 9). The length of resected segments is a critical factor impacting secure anastomoses. A cadaver study to determine the maximal limit of safe tracheal resection in humans reported that a 6.68 cm resection could be achieved safely (10). Grillo et al. reported that the maximum length of tracheal resection with safe anastomosis was 6.4 cm (11). However, individual variations, such as height, age, and gender, can significantly influence tracheal length. Therefore, tracheal rings of each patient instead of the absolute length was used to evaluate the defects in the current study, and we successfully performed nine tracheal ring resections with secure primary anastomoses in three cases, consistent with the largest defect reconstruction case previously reported (12).

Surgical release of the trachea and larynx, with blunt dissection of the trachea, cervical flexion, and suprahyoid release, have been crucial to achieve surgical success (13). Due to the large defects in the current study, a more extensive laryngeal release was performed by transection the suprahyoid and infrahyoid muscles, resection of the entire hyoid bone, and even cutting off the muscles and ligaments attached to the superior cornu of thyroid cartilage. Through a combination of these methods, all 16 patients in this study were successfully reconstructed, none with laryngeal dysfunction including alteration of voice, swallowing function and aspiration in long-term follow-up.

A continuous non-absorbable suture (“2-0” polypropylene) was conducted for the end-to-end tracheal anastomosis in the current study, which was different from the technique reported by Marulli (14). As reported by Marulli, tracheal anastomosis should be performed using a continuous absorbable suture (usually polydioxanone) for the membranous portion and interrupted absorbable stitches (usually polyglactin) for the cartilaginous one. Compared with interrupted stitches, our continuous suture technique can make the whole process of anastomosis visualized and is relatively simple. According to fibrolaryngoscope findings postoperatively in our previous cases, absorbable sutures (polydioxanone or polyglactin) were more likely to cause sputum retention compared with non-absorbable suture (polypropylene). Therefore, to reduce coughing due to sputum retention, polypropylene sutures were performed in the current study.

Tracheostomy is rarely necessary for short-segment resection as a tension-free end-to-end anastomosis can be performed (1). However, in the current study, a tension-free anastomosis was almost impossible to achieve in most patients due to the long-segment defects, despite adequate release of trachea and larynx. Therefore, a tracheostomy was performed for some patients to ensure the safety of airway. In some extreme cases when the trachea after anastomosis resided totally below the thoracic inlet, cricothyroidotomy offered a practical solution for airway protection.

Although sleeve resection with end-to-end anastomosis includes many advantages, limitations still exist. Surgical complications, anastomotic dehiscence with serious or even fatal outcomes have been reported (15). Additionally, the upper limit to the length of tracheal resection differs between individuals and cannot currently be precisely calculated before surgery. In extremely long-segment defect cases, a primary end-to-end anastomosis may not be possible even after adequate release of larynx and trachea. A primary reconstruction via appropriate flap with autologous or allogeneic graft support and tracheostomy followed by secondary reconstruction might be surgical alternatives (16, 17).

## 5 Conclusion

A primary end-to-end anastomosis following sleeve resection can be a practical and safe reconstruction strategy for long-segment defects exceeding six tracheal rings. Adequate release of the trachea and larynx are critically important for surgical success. Preventive tracheostomy or cricothyroidotomy may be necessary when the anastomosis is performed with high tension.

## Data Availability

The raw data supporting the conclusions of this article will be made available by the authors, without undue reservation.
